# Quality-Aware Signal Processing Mechanism of PPG Signal for Long-Term Heart Rate Monitoring †

**DOI:** 10.3390/s24123901

**Published:** 2024-06-16

**Authors:** Win-Ken Beh, Yu-Chia Yang, An-Yeu Wu

**Affiliations:** Graduate Institute of Electronics Engineering, National Taiwan University, Taipei City 10617, Taiwan; kane@access.ee.ntu.edu.tw (W.-K.B.); yvonne@access.ee.ntu.edu.tw (Y.-C.Y.)

**Keywords:** photoplethysmography, signal quality assessment, healthcare

## Abstract

Photoplethysmography (PPG) is widely utilized in wearable healthcare devices due to its convenient measurement capabilities. However, the unrestricted behavior of users often introduces artifacts into the PPG signal. As a result, signal processing and quality assessment play a crucial role in ensuring that the information contained in the signal can be effectively acquired and analyzed. Traditionally, researchers have discussed signal quality and processing algorithms separately, with individual algorithms developed to address specific artifacts. In this paper, we propose a quality-aware signal processing mechanism that evaluates incoming PPG signals using the signal quality index (SQI) and selects the appropriate processing method based on the SQI. Unlike conventional processing approaches, our proposed mechanism recommends processing algorithms based on the quality of each signal, offering an alternative option for designing signal processing flows. Furthermore, our mechanism achieves a favorable trade-off between accuracy and energy consumption, which are the key considerations in long-term heart rate monitoring.

## 1. Introduction

With the widespread use of wearable devices, photoplethysmography (PPG) has great potential for long-term vital sign monitoring to improve public health and alleviate the increasing obesity rate problem and medical burden. However, PPG is normally collected via wearable devices that are prone to suffer from artifacts. Signal corruption by artifacts will negatively influence measurement accuracy. With regards to this, PPG faces two major challenges in achieving long-term vital sign monitoring: (1) inaccurate vital monitoring caused by unstable signal quality, and (2) insufficient battery capacity for long-term monitoring.

**Figure 1:**
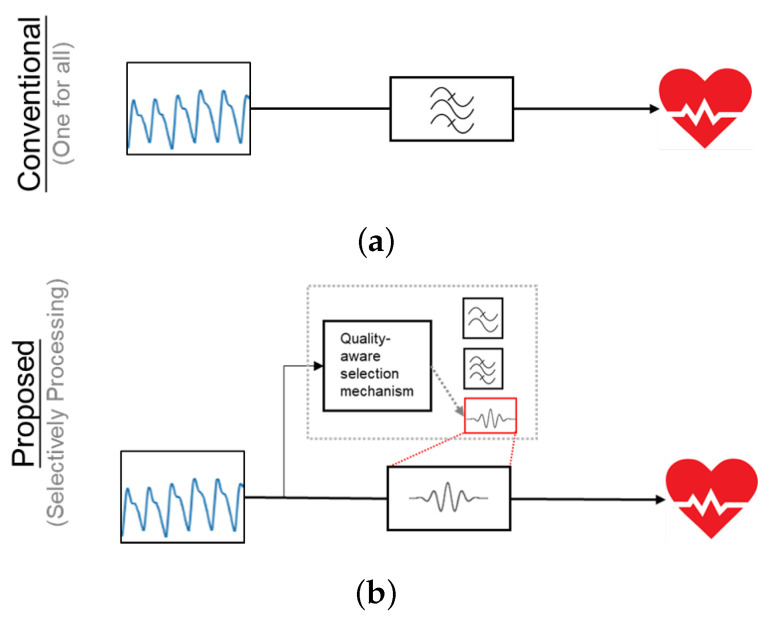
Comparison between (**a**) conventional processing flow that uses one algorithm for all signals, (**b**) proposed processing flow that selective chooses an algorithm based on signal quality.

Much PPG research focuses on optimizing processing algorithms to eliminate particular types of motion or noise artifacts [1,2,3,4,5] to ensure an accurate measurement. These artifacts are generated under different circumstances, which includes fast running [1,2,6], daily life activities [3,4], coughing [5], etc. However, in the long-term monitoring scenario, PPG signals could be collected under different circumstances at different times, which means the signal quality of collected PPG signals varies over time. Therefore, we suggest that selectively choosing a processing algorithm based on signal quality, which is illustrated in Figure 1, could bring benefit to the monitoring system in terms of accuracy and energy consumption, as [7] stated that a processing unit accounts for a large proportion (about 40%) of power consumption.

Conventionally, researchers discuss signal quality and processing algorithms separately. Some researchers have worked on developing or finding an optimal signal quality index (SQI) that represents or quantifies PPG’s quality [8,9,10] and have used them as features to distinguish clean and corrupted PPG segments through machine learning techniques [11,12,13,14]. However, few to no related works discuss the processing algorithm selection based on SQIs. We have only found that Zhang et al. [2] used kurtosis of the PPG spectrum from 0.8 Hz to 2.5 Hz, which is called the SQI, to ensure hand motions are reduced sufficiently, and then trigger the processing algorithm.

With regards to this, this paper presents a quality-aware processing mechanism of the PPG signal for long-term heart rate monitoring, which is an extension of [11]. The proposed mechanism evaluates incoming PPG signals by SQIs, and selectively chooses an algorithm for processing. We propose more options for the signal processing process in the proposed mechanism. When the incoming PPG signal has better quality, the proposed mechanism tends to select a lightweight processing algorithm. Therefore, we achieve higher efficiency by avoiding computation-intense algorithms while maintaining similar accuracy. The main contributions of this work are as follows:

(1.) To the best of our knowledge, we are the first to analyze the relation between PPG’s processing algorithms and an algorithm selection framework based on signal quality indices.
(2.) We present a novel quality-aware signal processing mechanism that selectively chooses algorithms based on incoming signal quality indices. The proposed mechanism enables a favorable trade-off between accuracy and energy consumption, which are the key considerations in long-term heart rate monitoring.

The next part of this paper is organized as follows. We will describe the background and motivation in Section 2. The proposed method will be presented in Section 3. In Section 4, we will present and discuss our experimental results. Finally, we present conclusions in Section 5.

## 2. Background

In the conventional signal processing flow, researchers tend to use robust algorithms to ensure the measurement system can adapt to high-noise scenarios. These powerful algorithms usually require much processing effort to ensure that the information contained in the signal can be acquired and analyzed. According to our observation, most of these robust algorithms work well in removing artifacts, thus achieving lower errors in the measurement. To investigate the effect of a particular algorithm, we have performed a simple experiment to show the differences between the “good” and “poor” algorithms.

### 2.1. Effect Comparison between Processing Algorithms

In this experiment, we initially analyze how the processing algorithm affects the PPG signals based on the case study of heart rate estimation.

**Figure 2:**
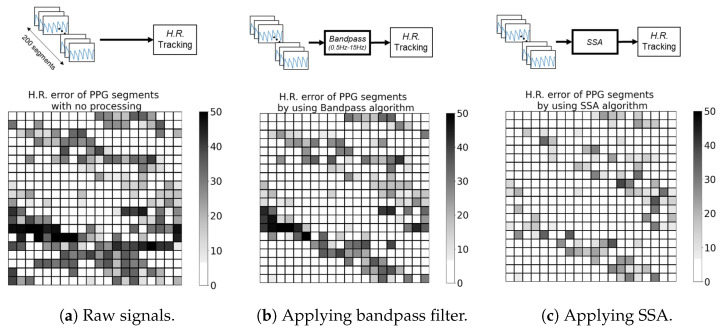
*H.R.* error heatmap of PPG segments with (**a**) no process, (**b**) applying bandpass (0.5–15 Hz) filter, (**c**) applying singular spectrum analysis, SSA. Each block represents the H.R. estimation error of a 10 s PPG signal. Corresponding *RMSE* of (**a**) 14.74 BPM, (**b**) 11.60 BPM, (**c**) 8.69 BPM.

- We took 400 10 s PPG segments for this experiment. These segments undergo different processing algorithms before performing heart rate estimation, which is spectral peak detection from the PPG spectrum between 0.83Hz and 2.16Hz.
- In Figure 2a, raw PPG segments are directly used in estimating H.R., while, in Figure 2b, we apply a bandpass filter (0.83 Hz–2.16 Hz), and singular spectrum analysis (SSA) in Figure 2c.

These three figures represent the signal processing flow with (a) no signal processing, (b) simple signal processing, and (c) intense signal processing, respectively. The grid matrices indicate the estimated *H.R.* error from the 400 PPG segments in different scenarios. As shown in Figure 2, these three grid matrices have different distributions of black spots. However, overall, the root mean square error (*RMSE*) of raw signals is greatest (14.74 BPM), the second is applying the bandpass filter (11.60 BPM), and the lowest (8.69 BPM) is applying the computational-intense processing algorithm, SSA.

### 2.2. Relation between Processing Algorithms

According to Figure 2, we did see some patterns between the error heatmaps of the three scenarios. To simplify the analysis, we said that the processing algorithm is sufficient for this PPG signal if the *H.R.* error of the processed PPG is less than 5 BPM. By setting up this rule, these gray-scale heatmaps turn into binary heatmaps. Therefore, we could make further analyses based on these maps.

**Figure 3:**
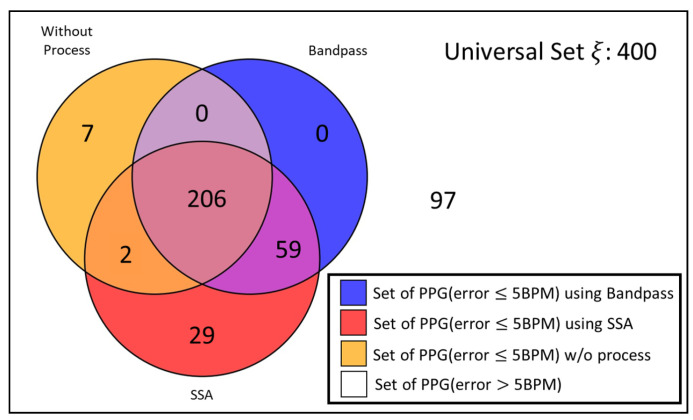
Venn diagram for describing the set of PPG segments, with an error ≤ 5 BPM, after applying processing algorithm.

As illustrated in Figure 3, we constructed a Venn diagram to visualize the effect of applying a processing algorithm and describe the relationship between each algorithm. Initially, there are 215 raw segments that can be used in estimating *H.R.*, which is framed by an orange circle in Figure 3. When the signals are applying a bandpass filter, the signal quality changes, and the circle of the Venn diagram moves and expands to the blue circle; this process makes the set bigger, increasing the high-quality signal from 215 to 265. It helps in improving signal quality, thus producing a lower error rate. However, there are several interesting points we can observe from this Venn diagram, which are:

1. Signal processing does not work for every signal; 7 out of 215 signals become worse if we apply a signal process (bandpass or singular spectrum analysis, SSA).
2. A computational-intense algorithm, like SSA, has a bigger set than the simpler algorithm. However, there is a significant intersection between these three sets, which have 206 signals. For those signals within the intersection, we could bypass the computational-intense algorithm if we selectively choose the processing algorithm.
3. The union of these three sets generates a bigger set. By selectively choosing an algorithm, we can improve overall accuracy theoretically.

With regards to this, we were motivated to create a quality-aware processing algorithm selection mechanism with a signal quality index (SQI).

## 3. Methodology

**Figure 4:**
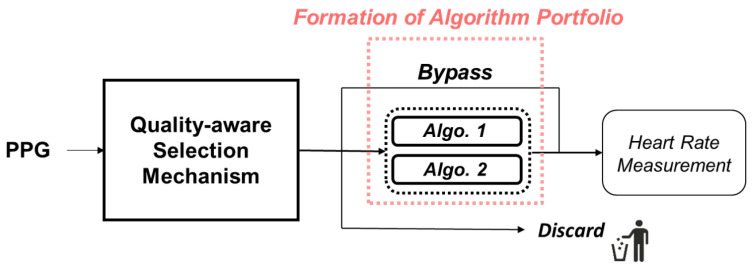
Schematic diagram of proposed mechanism. Proposed mechanism will choose most suitable algorithm from algorithm portfolio, which consists of three algorithms.

In this chapter, we will present an exposition of the operational principles of our mechanism. Our approach is based on the characteristic features of signal quality and involves selection among several processing algorithms. On the whole, our mechanism can be delineated into two main components, namely the formation of processing algorithm portfolio and the quality-aware selection mechanism, as illustrated in Figure 4. Subsequent sections will provide detailed descriptions of their respective implementation intricacies.

### 3.1. Formation of Processing Algorithm Portfolio

The formation of a processing algorithm portfolio assists us in selecting three algorithms from a pool of nine algorithms. The reasons for selecting algorithms from a larger algorithm pool to form the algorithm portfolio include:

- The limited data available for the training procedure make it challenging to train a classifier to select from a large algorithm pool. However, this task becomes more manageable when the number of algorithms is restricted to three.
- Some algorithms have a similar effect to others, and so selecting among these algorithms will become meaningless.

Hence, we will provide the guidelines for how we form the algorithm portfolio for the proposed quality-aware processing mechanism.

We surveyed several processing algorithms commonly used in PPG processing [1,15,16,17,18,19], which are shown in Table 1. These algorithms can be categorized into two groups, which are the static filtering method and the signal decomposition method.

#### 3.1.1. Similarity between Processing Algorithms

**Figure 5:**
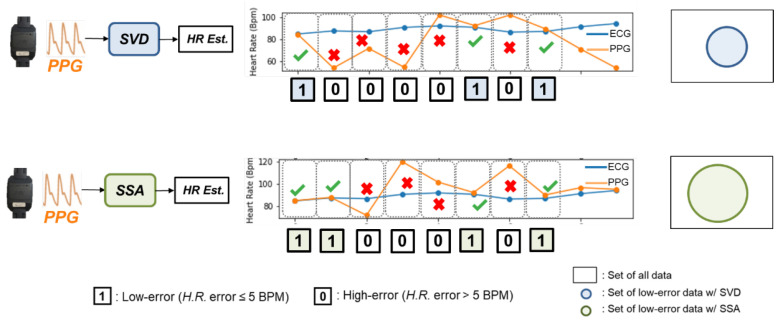
Schematic diagram showing the effect brought by different processing algorithms. We investigate the effect brought by different algorithms, and avoid selecting algorithms that have similar effects to the portfolio.

To avoid choosing processing algorithms with similar behavior, we must evaluate the similarity between different algorithms. Then we can remove those highly-correlated algorithms to achieve a smaller algorithm portfolio. To measure the similarity of the algorithms’ behaviors, we investigate the effect of each processing algorithm brought to the application’s outcome. Figure 5 shows the effect brought by the different processing algorithms. We have used different algorithms in this experiment, and we compare the outcome of each processed signal. After cross-checking with the reference H.R. calculated from an ECG, we can know which PPG segments can be processed by the algorithm. If the PPG segments have an estimated H.R. error lower than 5 BPM, the segment is denoted as “1”, which mean it is a high-quality signal (*H.R.* error ≤ 5 BPM) after processing. Otherwise, the data are labeled as “0”, as they produced a high-error result (*H.R.* error > 5 BPM).

**Figure 6:**
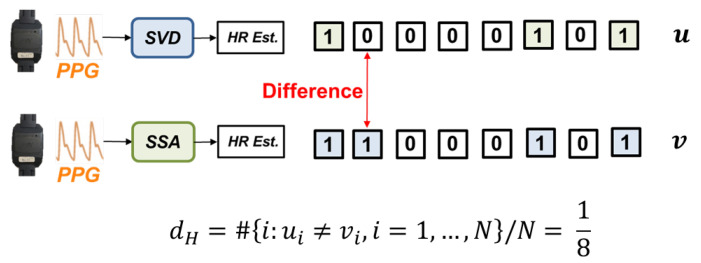
Schematic diagram of the calculation process of hamming distance.

Regarding these two binary sequences, we used hamming distance [20] to evaluate the similarity between the two sequences. The hamming distance, d(u,v), is defined as the number of places in which *u* and *v* differ, that is, $\#\{i:u_{i}\neq v_{i},i=1,\ldots ,n\}$. In the example of Figure 6, the hamming distance between the SVD and SSA algorithm equals 1, and then we normalize that to 1/8. If the calculated hamming distance is small, the two sequences are similar. That is, the difference in impact between these two processing algorithms is insignificant.

We continue this process to calculate the hamming distance between each pre-processing algorithm. The result is summarized in Table 2, which shows the hamming distance between each algorithm; a lower value of hamming distance indicates a higher similarity. For example, bypass and SSA have the greatest hamming distance, which is 0.198, indicating they have different impacts on the signal. On the other hand, LPF and bypass have a smaller hamming distance, indicating their impacts on the signal are similar.

#### 3.1.2. Formation of Processing Algorithm Portfolio

**Figure 7:**
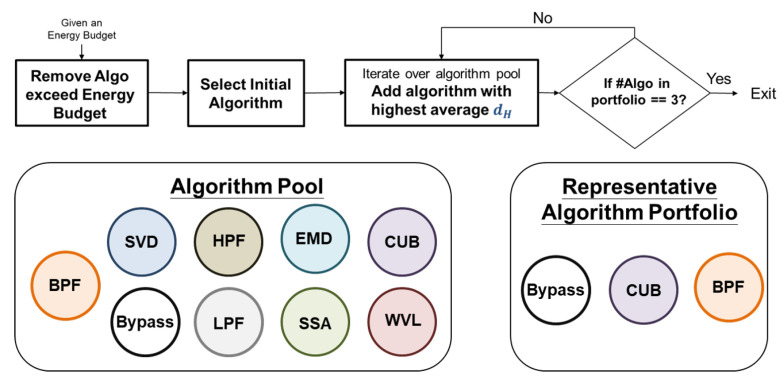
The procedure of selection method with hamming distance.

A diverse algorithm portfolio can provide more options for signal processing, thereby leading to improved results. Thus, we adopt a greedy approach to select an algorithm from the pool until the number of algorithms in the portfolio reaches three. The selection process is primarily based on the metric introduced earlier, namely, hamming distance. The process is illustrated in Figure 7 and comprises three steps:

(1.) Remove algorithms exceeding energy budget: given the energy constraint of every system, the initial step in forming the algorithm portfolio involves the elimination of algorithms that surpass the predetermined energy budget provided by the developer.
(2.) Select initial algorithm: we will select “bypass” as the initial algorithm in our framework due to its necessity as an option for preserving high-quality signals without processing. This feature could also contribute to energy conservation.
(3.) Iterate over the pool to select algorithms with highest average hamming distance: the next step is to iterate over the algorithm pool, and, for each candidate, we will compare it to the selected algorithm. We will compute the average hamming distance between the candidate and the selected algorithm, and select the candidate with the highest average hamming distance to be added to the portfolio.
(4.) Check if number of selected algorithms is equal to 3: terminate if the number of selected algorithms is equal to 3.

Choosing algorithms based on the hamming distance metric can lead to a more diverse algorithm portfolio, allowing for processing of a wider range of signals. To investigate this claim, we conducted an experiment. Firstly, we randomly selected algorithm combinations with different average hamming distances. Subsequently, we utilized our proposed quality-aware processing mechanism (to be introduced in the next chapter) to select the appropriate algorithm based on the incoming signal quality. The performance metric used to compare the different portfolios was the accuracy of heart rate estimation.

**Figure 8:**
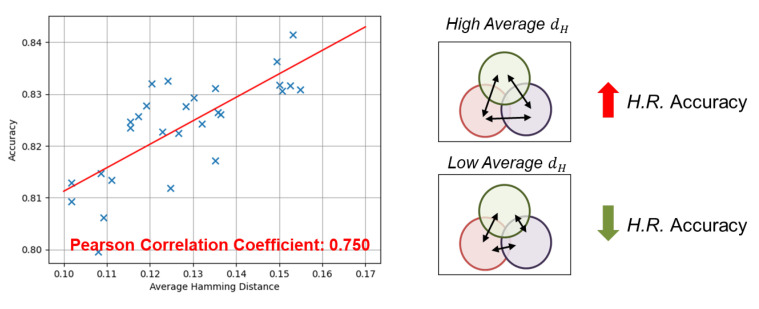
H.R. accuracy against average hamming distance between algorithms within same portfolio.

The results, as depicted in Figure 8, reveal a Pearson correlation coefficient of approximately 0.75 between the two variables, indicating a strong correlation [21]. This finding implies that the algorithm portfolio formation process can play a beneficial role in enhancing the performance of the proposed framework.

### 3.2. Quality-Aware Selection Mechanism

**Figure 9:**
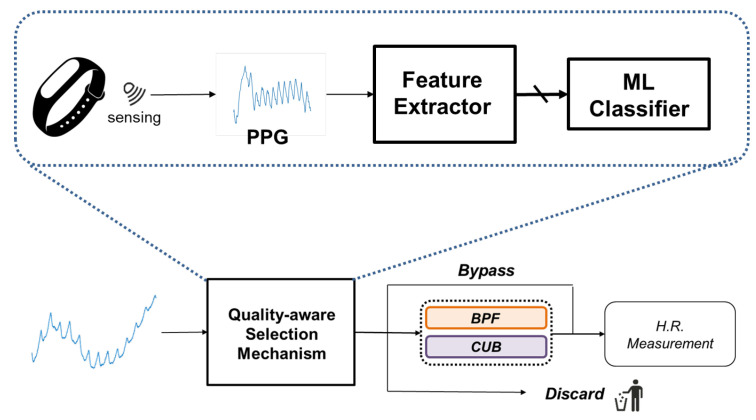
Overall flow of proposed quality-aware selection mechanism. Proposed mechanism evaluates and classifies incoming PPG signal quality, then selects the most suitable algorithm from the algorithm portfolio that consists of bypass, BPF, and CUB.

In this subsection, we present the implementation details of the quality-aware selection mechanism. The proposed mechanism is implemented as follows. Initially, an incoming PPG signal is passed through an feature extractor, which calculates features that represent the signal quality. Subsequently, these SQIs are input to a classifier that determines which algorithm should be used. The overall system architecture is depicted in Figure 9.

**Figure 10:**
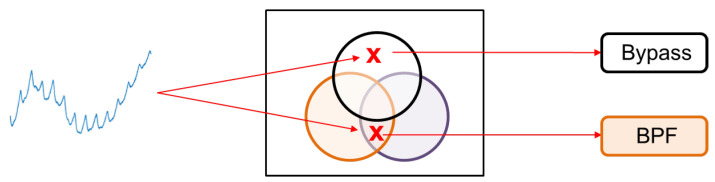
Illustration diagram of proposed mechanism by Venn diagram.

In order to demonstrate the proposed mechanism, we present a Venn diagram in Figure 10. The purpose of this diagram is to identify the region in which the incoming PPG signal belongs. Based on the region, the appropriate algorithm is recommended for processing.

#### 3.2.1. Feature Extraction

We extracted several low-complexity features for signal quality evaluation to achieve an efficient system. The SQIs we used are summarized in Table 3. These features include statistical features and frequency-domain features. Statistical features include median, standard deviation (STD), kurtosis, skewness, and entropy. They are commonly used to denote the statistical properties of the signal in the time domain. Frequency-domain features, such as the standard deviation of the power spectrum and the total power at specific frequency intervals, reflect the power of the incoming signals. We can observe the frequency components to determine whether the signal is affected by noise.

#### 3.2.2. Classifier Design

**Figure 11:**
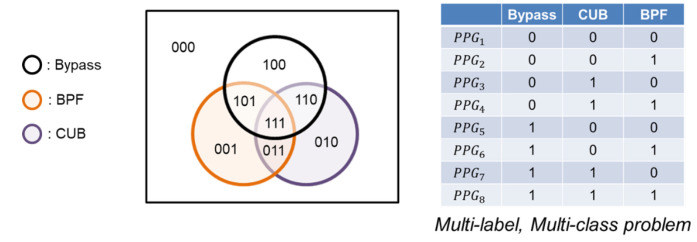
Each PPG is eligible for several processing algorithms.

**Figure 12:**
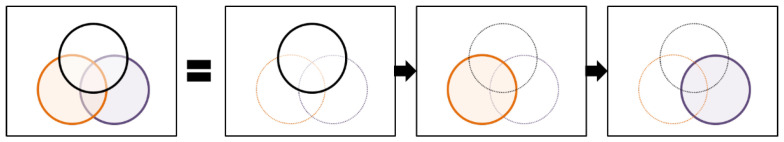
Schematic diagram of classification problem simplification.

The present classification problem involves the determination of which region in the Venn diagram an incoming PPG signal belongs to, which constitutes a multi-label, multi-class problem. Specifically, there are several options for each PPG signal, thereby resulting in a multi-class problem. At the same time, each PPG signal is eligible for several options, as indicated by correct signs in multiple columns, resulting in a multi-label problem. These aspects are illustrated in Figure 11. Therefore, we have made some designs on the ML classifier for this multi-class, multi-label problem, which are:

1. Cascade classifier: To address the multi-class, multi-label problem, we simplify the task by breaking it down into a cascade of binary classification problems. As depicted in Figure 12, the cascade binary classification approach begins by determining if the signal is eligible for bypass, followed by checks for BPF and CUB, respectively. If none of these algorithms are deemed suitable for effective processing, the signal is discarded.

**Figure 13:**
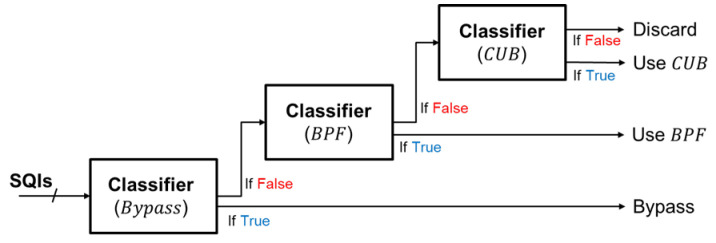
Illustration diagram of cascade classifier.

Therefore, a cascade of three XGBoost (eXtreme Gradient Boosting) [22,23] binary classifiers is employed to assist in determining the appropriate algorithm for processing the incoming signal. The cascade classifier is depicted in Figure 13. The extracted SQIs are first fed into the initial classifier, which determines whether Algorithm 1 is suitable for the signal. If the classifier output is true, we immediately perform processing using bypass. Conversely, if bypass is not recommended by the classifier, we proceed to the second classifier to check whether BPF is a better fit for the signal. If none of the three classifiers recommend any algorithm for the incoming signal, it is discarded to avoid potentially high error estimation.

**Figure 14:**
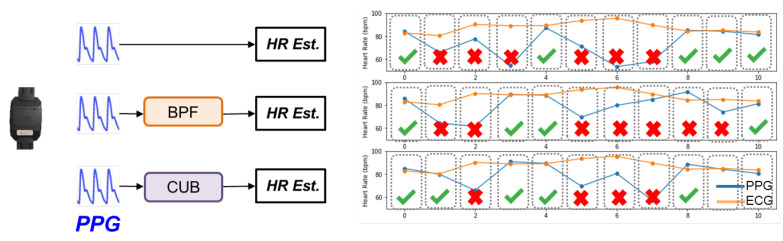
Process of acquiring label for training XGBoost classifier.

2. Training flow: Initially, we divide the input signals into non-overlapping 10 s segments. Subsequently, we extract 14 signal quality features from the raw PPG signals, along with the labels annotated by ECG signals, to train the classifier. As illustrated in Figure 14, we take BPF as an example and assign the label 1 if a signal is eligible for BPF; otherwise, it is assigned 0.

**Figure 15:**
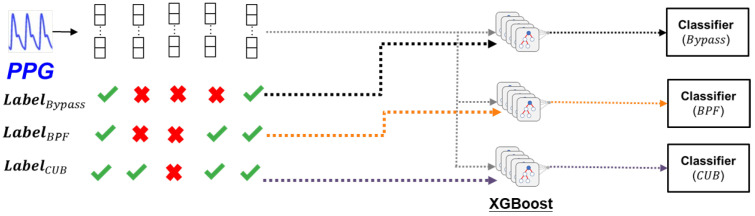
Training stage of three XGBoost classifiers to distinguish the data usability under bypass, BPF, and CUB.

After collecting the labels for bypass, BPF, and CUB, we proceed to train the XGBoost classifier. Figure 15 depicts the training stage of the XGBoost classifier, where we set it as a binary classifier and use the logistic loss function for model learning. We utilize grid search to find the optimal parameters for XGBoost to achieve the best performance in distinguishing data usability under bypass, BPF, and CUB processing. Finally, we obtain three binary classifiers, which we cascade to perform algorithm selection from a portfolio that contains three algorithms.

**Figure 16:**
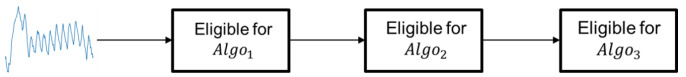
Illustration diagram of determining the order of binary classifier.

3. Classifier order rearrangement: The arrangement of these binary classifiers is deemed to be a significant factor influencing the overall performance. The inquiry regarding the optimal arrangement of these classifiers, depicted in Figure 16, dictates the order in which the classifiers are to be placed in the first, second, and third stages. This subsection aims to demonstrate the impact of reordering the binary classifiers and presents the proposed reordering strategy.

**Figure 17:**
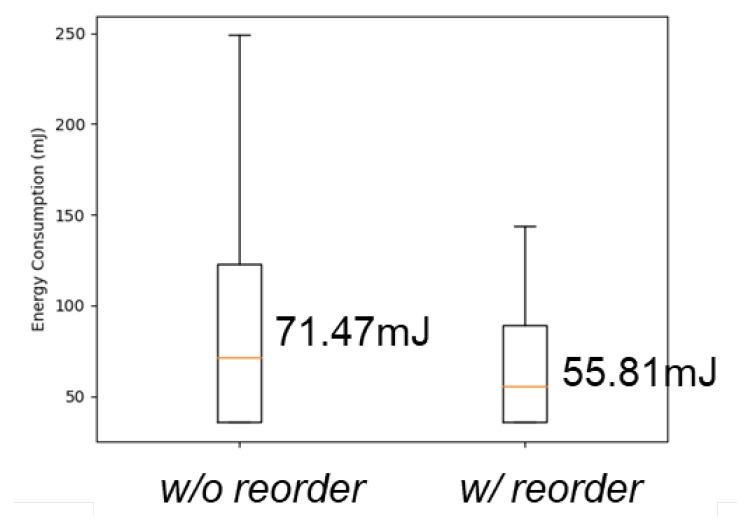
Box plot of energy consumption between classifiers with and without reorder by algorithm’s energy consumption.

As the classifiers operate sequentially, it is possible for the signal to exit early from the first and second classifiers. A desirable outcome would involve the signal being processed by an algorithm with reduced energy consumption. In the event that the signal is deemed suitable for algorithm 1, early exit from the first or second classifier would aid in conserving energy consumption for the entire system.
In light of this perspective, algorithm 1 should represent a processing method with comparably lower energy consumption, while algorithm 2 should have the second lowest energy consumption, and algorithm 3 should exhibit the highest energy consumption. For instance, the CUB, BPF, and bypass portfolio can be rearranged in the order of bypass, BPF, and CUB. Through this rearrangement of algorithms, it is feasible to achieve reduced energy consumption.
Subsequently, a straightforward experiment was conducted to demonstrate the impact of reordering the binary classifiers. Two scenarios were compared based on energy consumption, namely: (1) the classifiers without being reordered by algorithm energy consumption, and (2) the classifiers reordered based on energy consumption. The experimental outcomes are displayed in Figure 17, and suggest that the reordering of the binary classifiers based on the algorithm’s energy consumption can lead to a general reduction in energy consumption.

## 4. Experiment Results

### 4.1. Experiment Setup

#### 4.1.1. Dataset

For long-term PPG monitoring, the dataset contains two-channel PPG signals and a channel ECG signal in each subject (11 males and 1 female, ages 22 to 25). PPG signals are recorded from wrist-type PPG sensors with a green LED. ECG signals are recorded from the chest using Procomp Infiniti. The sampling rates of PPG and ECG are at 100 Hz and 256 Hz, respectively.

**Figure 18:**
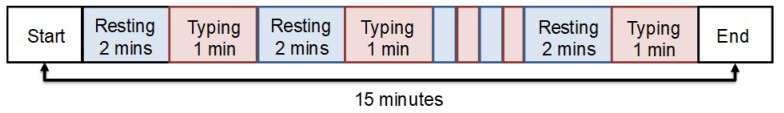
Experimental flow of the data collection.

To generate some artifacts or corruption to the signals, subjects were asked to type the same article during the collection of wrist PPG. The experiment flow is shown in Figure 18. At the beginning of the experiments, subjects were requested to be static for 2 min. Afterward, they typed the article for 1 min. This process was repeated five times, and the total length of the experiment was 15 min. Due to the constraints of our experimental equipment, we were unable to record wireless ECG signals during general movement scenarios. Consequently, we confined our experiment to static conditions.

#### 4.1.2. Energy Consumption Estimation

To assess the energy consumption of each algorithm, we employed the RAPL (Running Average Power Limit) power meter. RAPL is a power management technology that permits the CPU to reallocate workload between CPU cores [24]. It includes a power meter that can measure the energy usage of a host machine and the execution of certain programs. Previous studies have demonstrated that RAPL’s estimates are highly correlated with actual power consumption [25,26]. We present the energy consumption of each algorithm in Table 4.

### 4.2. Selected Portfolio Analysis

Once we have considered the energy budget, we take out the algorithm that exceeds the energy budget from the algorithm pool. Then, we perform the portfolio formation process. We summarize the portfolio under different energy constraints in Table 5.

**Figure 19:**
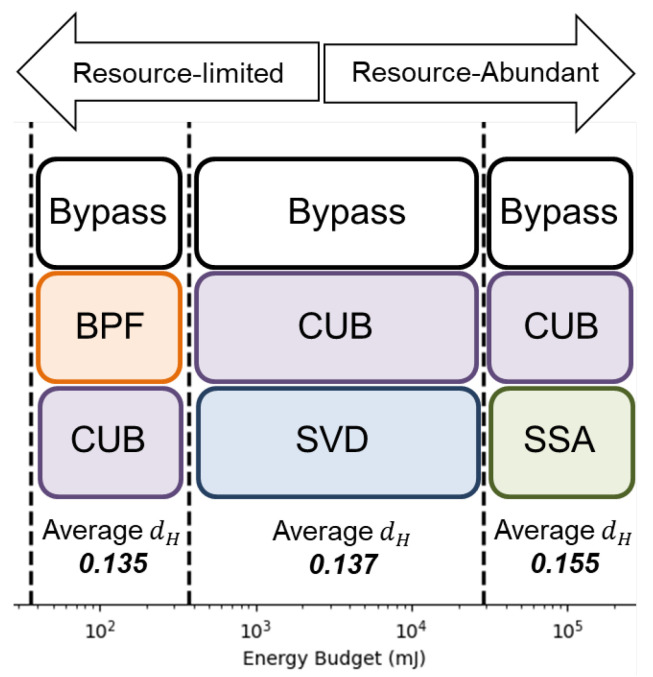
Different algorithm portfolios under resource-limited and resource-abundant scenarios.

Table 5 displays the variation in the selected algorithms within the portfolio under different energy budgets. Notably, the selected algorithms differ depending on the available energy budget. For an energy budget below 35.54 mJ, our proposed quality-aware processing mechanism is not recommended due to its energy overhead. In such a case, the one-for-all processing mechanism is preferred. However, for higher energy budgets, the portfolio is inclined towards using more powerful, yet energy-hungry, algorithms. This highlights the flexibility offered by the proposed algorithm portfolio formation in enabling developers to choose algorithms based on energy budgets. The selected portfolios under resource-limited and resource-abundant scenarios are summarized in Figure 19.

### 4.3. Comparison between Frameworks

In this subsection, we will make comparisons between frameworks. We will compare them in terms of accuracy, mean absolute error (MAE), and consumed energy. The frameworks included for comparison are as follows. (1.) one-for-all (OFA) processing, using WVL and SSA as processing algorithms. (2.) Signal quality assessment (SQA): the signal will be assessed by SQA, and poor-quality signals will be discarded for better measurement results. (3.) Quality-aware processing (QAP) with the portfolio of bypass, BPF, and CUB. The comparisons are summarized in Table 6.

Initially, it can be observed that both the SQA and QAP mechanisms rejected 30% of signals for H.R. measurement, which were deemed to be of poor quality. The reject rate of 30% was deliberately chosen as a parameter in our experimental settings. The reason we chose the 30% of rejection rate was because

1. With a 30% rejected signal, we can achieve around an 80% H.R. accuracy rate, which can provide the wearable device user with a satisfactory experience.
2. Meanwhile, the 30% missing heart rate signals can be recovered through interpolation methods or some missing data imputation methods. The study by [27] demonstrates the impact of different percentages of missing values on HRV-related features in the presence of motion artifacts. With 30% missing values, interpolated results have a relative error that is less than 10%.

**Figure 20:**
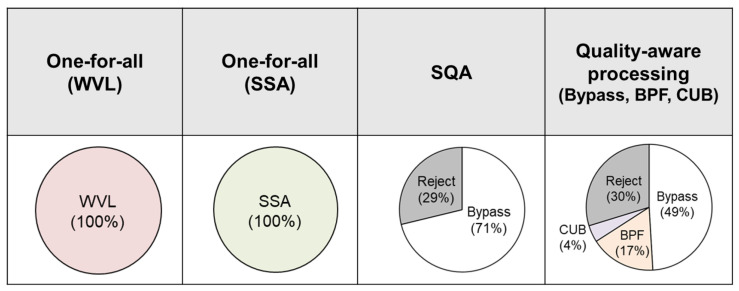
Comparison of selected pre-processing algorithms between frameworks.

The experiment result indicated the OFA processing scheme did not discard any signals. The key disparity between SQA and QAP lies in the fact that QAP permits signals to undergo various methods. As per Figure 20, 49% of signals bypass the processing, 17% of them undergo BPF, and 4% of them undergo CUB. The QAP framework, which offers greater options in terms of processing methods, achieved higher accuracy compared with both SQA and OFA frameworks, while discarding the same proportion of signals.

Apart from accuracy, it is noteworthy that the use of SSA as a processing algorithm in the OFA algorithm results in significant energy consumption. Conversely, the utilization of WVL as a processing algorithm results in lower energy consumption, but with a compromise in terms of accuracy.

Based on our observations, we can infer that if we have the option to discard poor-quality signals, there is no need to design energy-intensive processing methods to achieve higher accuracy. Instead, we can focus on processing signals with moderate quality. Consequently, both SQA and QAP frameworks consume less energy compared with using only SSA.

In terms of overhead, the QAP framework typically involves calculating SQIs. Since the SQIs utilized in our study are of low complexity, the resulting energy consumption overhead is minimal (+35.536 mJ) compared with OFA. Nevertheless, we achieve higher accuracy and a lower mean absolute error (MAE).

In comparison with OFA (WVL), we achieve an 8.7% accuracy improvement with an additional energy consumption of 28.07 mJ per photoplethysmography (PPG) segment. Conversely, in comparison with OFA (SSA), we achieve a 3.0% accuracy improvement while consuming only 0.1% of the energy consumption of OFA (SSA).

### 4.4. Analysis on Signals Passing Each Stage

In this subsection, we will give more detailed analysis on the signal passing each classifier. Our experiment results are based on the leave-one-subject-out (LOSO) setting.

**Figure 21:**
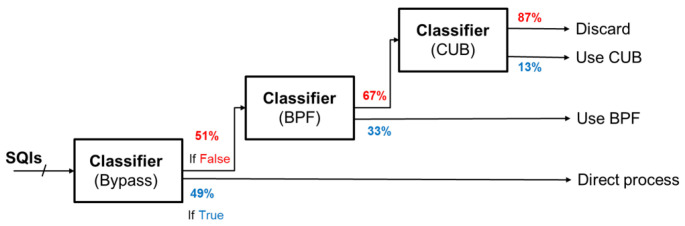
The portion of signals passing each classifier.

As illustrated in Figure 21, the proposed mechanism consists of three cascaded classifiers. The figure presents the percentage of signals that passed through each classifier. For instance, approximately 49% of signals were recommended to bypass in the first stage, while, in the second stage, 33% of the remaining signals were recommended to undergo BPF as the processing algorithm. Based on this distribution, we can classify these signals into four distinct groups.

1. Group I: signals that suggested bypass;
2. Group II: signals that suggested BPF;
3. Group III: signals that suggested CUB;
4. Group IV: Poor quality signal that requires discarding.

Next, we conducted an analysis of signals from different groups. Initially, we assessed the quality of signals in Group I, which we advise not to process, and found that they generally exhibit higher quality. Additionally, we compared the original accuracy of each group without processing. Notably, Group I, which we recommend bypassing processing, exhibited the highest signal quality, followed by Group II, Group III, and finally Group IV, which we recommend discarding due to its low original accuracy. The results are summarized in Table 7.

These findings suggest that the proposed mechanism appropriately recommends algorithms based on incoming signal quality. Specifically, we recommend that high-quality signals should not undergo processing, signals of moderate quality should be subjected to simple algorithms (e.g., bandpass filtering), and poor-quality signals should be processed using powerful, energy-intensive algorithm methods. Additionally, we recommend that signals deemed unsalvageable by the current processing approach be discarded to avoid adversely affecting subsequent measurements.

### 4.5. Limitations

This study faces several limitations. First, the contaminated data generated are solely from typing actions, introducing a limited variety of noise sources. Second, the absence of precise timestamps marking typing actions hinders accurate analysis and evaluation of the typing data’s rejection rate. Without these timestamps, determining when PPG signals transition into a typing state is challenging, complicating the assessment of signal quality. With regards to this, we will refine our experimental setup in future research to include a broader array of noise sources and precise temporal markers to ensure a more detailed and accurate analysis.

## 5. Conclusions

We presented a quality-aware signal processing mechanism for the application of long-term heart rate monitoring. In comparison with OFA (WVL), we achieve an 8.7% accuracy improvement with an additional energy consumption of 28.07 mJ. Conversely, in comparison with OFA (SSA), we achieve a 3.0% accuracy improvement while consuming only 0.1% of the energy consumption of OFA (SSA). The experiment results indicate selectively choosing an algorithm based on signal quality could benefit a good accuracy–energy tradeoff, which fits the application of a long-term monitoring scenario. 

## Figures and Tables

**Table 1:** Settings for processing algorithm implementation.

Category	Algorithm	Description
Filtering	Bandpass Filter	5th order Butterworth (0.5–15 Hz)
	Low-pass Filter	5th order Butterworth (2.5 Hz)
	High-pass Filter	5th order Butterworth (0.5 Hz)
Decompose	Cubic Spline Interpolate [15]	-
	Wavelet Filtering [16]	Daubechies 8 (db8)
	SVD of T-F Distribution (SVDTFD) [17]	First and second components
	Empirical Mode Decomposition (EMD) [18]	Remove EMD components 0.25 Hz
	Singular Spectrum Analysis (SSA) [1,19]	Remove SSA components 1 Hz

**Table 2:** Hamming distance between each algorithm, where a lower hamming distance between algorithms means higher similarity.

${\mathrm{Alg}}_{1}$	-	BPF	EMD	CUB	SSA	WVL	SVD	HPF
${\mathrm{Alg}}_{2}$								
LPF	0.001	0.162	0.152	0.163	0.197	0.152	0.166	0.161
HPF	0.162	0.021	0.059	0.080	0.090	0.037	0.040	
SVD	0.167	0.032	0.066	0.079	0.095	0.053		
WVL	0.180	0.042	0.041	0.074	0.097			
SSA	0.198	0.091	0.107	0.103				
CUB	0.164	0.079	0.091					
EMD	0.153	0.064						
BPF	0.163							

**Table 3:** All candidate features.

Feature Type	Features
Statistics Features	Median
	Range
	Standard Deviation
	Kurtosis
	Skewness
	Entropy
Frequency Domain Features	PSD in {1 Hz, 3 Hz, 5 Hz, 7 Hz, 0.01–1 Hz, 1–3 Hz}
	PSD Ratio of {1–3 Hz/0.01–1 Hz}
	STD of Frequency Spectrum

**Table 4:** Energy consumption of each algorithm estimated by RAPL.

Algo.	Energy Consumption (mJ)
Bypass	0
Bandpass Filter (BPF)	10.05
Empirical Mode Decomposition (EMD)	2268.50
Cubic Spline Interpolation (CUB)	10.85
Singular Spectrum Analysis (SSA)	28,767.42
Wavelet Decomposition (WVL)	9.73
Singular Value Decomposition (SVD)	333.03
High-pass Filter (HPF)	14.57
Low-pass Filter (LPF)	10.28

**Table 5:** Algorithm portfolios under different energy budgets.

Energy Budget (mJ)	Selected Portfolio		
E < 35.54	One-For-All processing		
35.54 ≤ E < 368.57	Bypass	CUB	BPF
368.57 ≤ E < 28,802.96	Bypass	CUB	SVD
E ≥ 28,802.96	Bypass	CUB	SSA

**Table 6:** Comparisons between frameworks in the application of heart rate monitoring.

	One-for-All (WVL)	One-for-All (SSA)	SQA	QAP (Bypass, BPF, CUB)
H.R. Accuracy (%)	74.5	80.2	77.8 ± 13.8	83.2 ± 11.0
Mean Absolute Error (BPM)	6.02 ± 10.3	4.1 ± 7.6	6.1 ± 10.7	4.7 ± 9.2
Per sample Energy Consumption (mJ)	9.73	28,767.4	35.5	37.8

**Table 7:** The accuracy of each signal group without processing in proposed mechanism.

	Accuracy w/o Process	MAE w/o Process
Group I	0.84	4.42 ± 8.82
Group II	0.63	9.96 ± 13.86
Group III	0.62	8.37 ± 10.85
Group IV	0.37	14.70 ± 12.79

## Data Availability

The raw data supporting the conclusions of this article will be made available by the authors on request.
